# Sexually Transmitted Infection Treatment Rates Among Pregnant vs Nonpregnant Patients in Emergency Departments

**DOI:** 10.1001/jamanetworkopen.2026.4911

**Published:** 2026-04-15

**Authors:** Michael Gottlieb, Eric Moyer, Giles W. Slocum, Melissa Fleegler, Richard E. Rothman, Yu-Hsiang Hsieh, Supriya D. Mehta, Kyle J. Popovich, Jason Haukoos

**Affiliations:** 1 Department of Emergency Medicine, Rush University Medical Center, Chicago, Illinois; 2 Department of Emergency Medicine, University of New Mexico Health Sciences Center, Albuquerque; 3 Department of Emergency Medicine, The Johns Hopkins University, Baltimore, Maryland; 4 Division of Infectious Diseases, Department of Medicine, Rush University Medical Center, Chicago, Illinois; 5 Department of Emergency Medicine, Denver Health, Denver, Colorado; 6 Department of Emergency Medicine, University of Colorado School of Medicine, Aurora; 7 Department of Epidemiology, Colorado School of Public Health, Aurora

## Abstract

**Importance:**

Emergency departments (EDs) play a central role in the evaluation and treatment of sexually transmitted infections, particularly for patients with limited access to outpatient care. National guidelines recommend empiric treatment for *Neisseria gonorrhea* and *Chlamydia trachomatis* when clinical concern is present or follow-up cannot be ensured. However, there are limited data on how empiric treatment is applied across pregnancy status and patient demographics in the ED setting.

**Objective:**

To compare empiric treatment rates between pregnant and nonpregnant patients evaluated for *N gonorrhea* and *C trachomatis* infection in EDs and to examine demographic differences in empiric treatment within these groups.

**Design, Setting, and Participants:**

Repeated cross-sectional study using aggregate encounter-level data from Epic Cosmos, a national deidentified electronic health record database, from January 1, 2016, to December 31, 2024, of ED patients for whom *N gonorrhea* or *C trachomatis* testing was performed. Data were analyzed from January 21 to 25, 2026.

**Exposure:**

Pregnancy status at the time of the ED encounter.

**Main Outcomes and Measures:**

The primary outcome was empiric treatment, defined as administration of a guideline-recommended antibiotic regimen for *N gonorrhea* or *C trachomatis* infection during the same ED encounter as the testing. Associations between patient characteristics and empiric treatment were estimated using unadjusted odds ratios with 95% CIs, calculated from aggregate counts.

**Results:**

Among 4 904 343 ED encounters with *N gonorrhea* or *C trachomatis* testing, 49 419 of 454 048 pregnant patients (10.9%) and 1 699 393 of 4 450 295 nonpregnant patients (38.2%) received empiric treatment. Pregnant patients had substantially lower odds of empiric treatment than nonpregnant patients (odds ratio, 0.20; 95% CI, 0.20-0.20). For both pregnant and nonpregnant patients, empiric treatment was more common among younger patients, those with noncommercial insurance, and patients whose primary language was English. Differences by race and ethnicity were observed but varied by pregnancy status.

**Conclusions and Relevance:**

These findings suggest that in a national ED sample, empiric treatment for patients tested for *N gonorrhea* and *C trachomatis* differed markedly by pregnancy status and across demographic characteristics. These differences may reflect a combination of guideline interpretation, communication, and structural barriers to follow-up rather than uniform clinician bias. Understanding how empiric treatment aligns with population-level risk and access to care may inform more equitable management of sexually transmitted infections in ED settings.

## Key Points

**Question:**

Do empiric treatment rates for patients tested for *Neisseria gonorrhea* and *Chlamydia trachomatis* infection in emergency departments differ by pregnancy status, and are these differences associated with patient demographic characteristics?

**Findings:**

In this cross-sectional study of more than 4.9 million emergency department encounters with *N gonorrhea* or *C trachomatis* testing, empiric treatment occurred in approximately 11% of pregnant patients compared with 38% of nonpregnant patients. Among pregnant patients, empiric treatment rates differed by age, insurance status, language, race, and ethnicity.

**Meaning:**

These findings suggest that differences were observed in empiric sexually transmitted infection treatment by pregnancy status and demographic characteristics, and these patterns may reflect a combination of guideline interpretation, communication, and structural barriers to follow-up rather than uniform inequity, emphasizing the need for context-aware approaches to equitable care.

## Teaser

This cross-sectional study compares empiric treatment rates between pregnant and nonpregnant patients evaluated for *Neisseria gonorrhea* and *Chlamydia trachomatis* in emergency departments and examines demographic differences in empiric treatment within these groups.

## Introduction

Sexually transmitted infections (STIs) continue to pose a substantial public health burden in the United States, with *Neisseria gonorrhea* and *Chlamydia trachomatis* infections accounting for approximately 6.4 million new infections annually.^1^ *N gonorrhea* and *C trachomatis* infections are associated with substantial morbidity, including pelvic inflammatory disease, infertility, ectopic pregnancy, and adverse neonatal outcomes.^2,3,4^ Despite advances in screening and treatment, the incidence of STI has increased over the past decade, reflecting persistent gaps in prevention, access to care, and timely treatment.^5,6^

Emergency departments (EDs) occupy a critical position in the STI care continuum. For many individuals, particularly those facing barriers related to insurance status, housing instability, and limited access to primary care, the ED serves as the primary or sole point of health care contact.^7,8,9,10,11,12^ In this setting, clinicians must often make treatment decisions in the context of diagnostic uncertainty, time pressure, and limited ability to ensure follow-up.^13^ Recognizing these challenges, the Centers for Disease Control and Prevention have recommended empiric treatment for *N gonorrhea* and *C trachomatis* when clinical concern exists and follow-up cannot be reliably ensured.^14^ Importantly, these recommendations intentionally lower the threshold for treatment in settings and populations where structural barriers make delayed treatment more likely to result in harm.

Pregnancy introduces further complexity into STI evaluation and management in the ED setting. Untreated *N gonorrhea* and *C trachomatis* infections during pregnancy are associated with increased risks of preterm birth, low birth weight, neonatal conjunctivitis, and neonatal pneumonia.^15,16^ However, overuse of antibiotics increases the risk of complications, including allergic reactions, antibiotic-associated infections (eg, candidiasis), and antibiotic resistance.^13^ Clinicians may exercise increased caution with empiric antibiotic use during pregnancy because of concerns about fetal safety, diagnostic uncertainty related to overlapping genitourinary symptoms, and the expectation that pregnant patients may have improved follow-up through prenatal care.^17^ How these competing considerations influence empiric treatment decisions in EDs has not been well characterized at a national level.

Prior studies examining STI care in EDs have identified variations in treatment rates by sex, with higher rates of empiric treatment for men.^18^ Data on other demographic factors are more limited, although some have also identified differences by race.^19^ These studies were limited by small sample sizes, selected demographic characteristics, and limited contextualization of surrounding factors that may influence treatment decisions. Therefore, there is a need to better understand treatment differences across pregnancy status and demographic groups to inform the current understanding and opportunities for improving treatment approaches in the ED setting. The objective of this study was to compare empiric treatment rates between pregnant and nonpregnant patients evaluated for *N gonorrhea* or *C trachomatis* infection in EDs and describe how empiric treatment varies across demographic characteristics within these groups.

## Methods

### Study Design

We performed a repeated cross-sectional study using Epic Cosmos, a deidentified electronic health record (EHR) database aggregating data from more than 43 000 hospitals and clinics. Epic Cosmos includes more than 300 million unique patients and is broadly representative of the US Census with respect to age, race, ethnicity, and insurance coverage.^20^ Data are provided in aggregate form, enabling large-scale descriptive analyses while preserving patient privacy. This study followed the Strengthening the Reporting of Observational Studies in Epidemiology (STROBE) reporting guideline for cross-sectional studies.^21^ The Rush University institutional review board deemed the study to be exempt from review and informed consent because it did not use human participants.

### Study Population

We included all ED encounters for patients aged 15 years or older from January 1, 2016, through December 31, 2024, in which testing for *N gonorrhea* or *C trachomatis* was performed. Patients younger than 15 years were excluded because treatment decision-making for suspected STIs in this population can differ systematically from that of older adolescents and adults. Pediatric management often involves age-specific clinical pathways, nuanced weight-based dosing, and specific antimicrobial safety considerations (eg, avoidance of tetracyclines in younger cohorts). Inclusion of this group in a cross-sectional analysis could introduce heterogeneity and confounding, limiting comparability of empiric antibiotic use between groups. Encounters were categorized by pregnancy status at the time of the ED visit, as documented in the EHR.

### Variables

Patient characteristics examined included age, sex, race, ethnicity, primary language, and payer source. Categories of sex were male and female, as documented in the EHR, and reflect sex assigned at birth. Data on gender identity were not consistently available. Encounters with missing data or other or not reported sex were included in overall descriptive analyses but excluded from pregnancy-specific comparisons. Race and ethnicity were obtained from the EHR and based on patient self-report. Race categories included American Indian or Alaskan Native, Asian, Black or African American, Native Hawaiian or Other Pacific Islander, White, other race, or not reported. Other race is a category in Cosmos and is not further defined. Ethnicity was categorized as Hispanic or Latino, not Hispanic or Latino, or not reported. Primary language was categorized as English, Spanish, other language or not reported. Payer source was categorized as commercial or other insurance, Medicaid, Medicare, not reported, or self-pay.

### Outcome

The primary outcome was empiric treatment for *N gonorrhea* or *C trachomatis* infection, defined as administration of a Centers for Disease Control and Prevention–recommended antibiotic (eg, ceftriaxone, azithromycin, or doxycycline) during the same encounter in which STI testing was performed.^14^ We also performed a subgroup analysis comparing empiric antibiotic administration between pregnant and nonpregnant female patients.

### Statistical Analysis

Because Epic Cosmos data are available only in aggregate form, patient-level multivariable modeling was not possible. We calculated empiric treatment rates overall and stratified by pregnancy status and demographic characteristics. Associations between patient characteristics and empiric treatment were estimated using unadjusted odds ratios (ORs) with 95% CIs, calculated from aggregate counts. These ORs describe relative differences in empiric treatment rates and do not account for potential confounding by correlated demographic or clinical factors. All statistical tests were 2-sided, with a significance threshold of *P* < .05. Analyses were performed using IBM SPSS, version 31.0.0.0. Data were analyzed from January 21 to 25, 2026.

## Results

### Study Population

Among 320 742 155 ED encounters during the study period, 4 904 343 (1.5%) involved patients who received testing for *N gonorrhea* or *C trachomatis*. Of these encounters, 454 048 (9.3%) involved pregnant patients, and 4 450 296 (91.7%) involved nonpregnant patients.

### Empiric Treatment by Pregnancy Status

**Figure 1.**
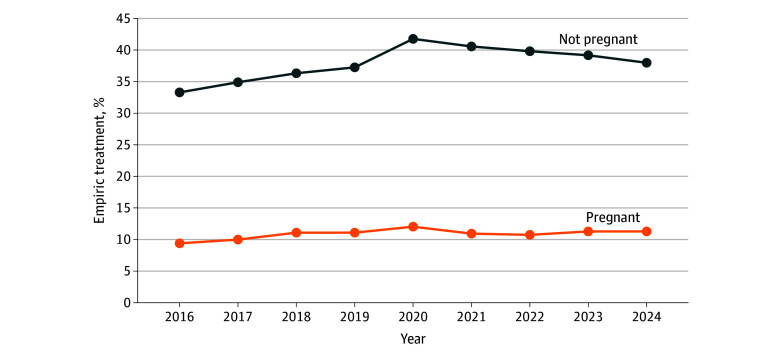
Line Graph of Rate of Empiric Treatment Among Emergency Department Patient Encounters Receiving Testing for *Neisseria gonorrhea* or *Chlamydia trachomatis*

Overall, 10.9% of pregnant patients (49 419 of 454 048) and 38.2% of nonpregnant patients (1 699 393 of 4 450 295) received empiric treatment during their ED encounter (Figure 1). Pregnant patients had substantially lower odds of empiric treatment compared with nonpregnant patients (OR, 0.20; 95% CI, 0.20-0.20). In a subgroup analysis comparing female patients only, pregnant patients had significantly lower odds of receiving empiric treatment compared with nonpregnant patients (OR, 0.27; 95% CI, 0.27-0.28).

### Demographic Differences Among Pregnant Patients

**Figure 2.**
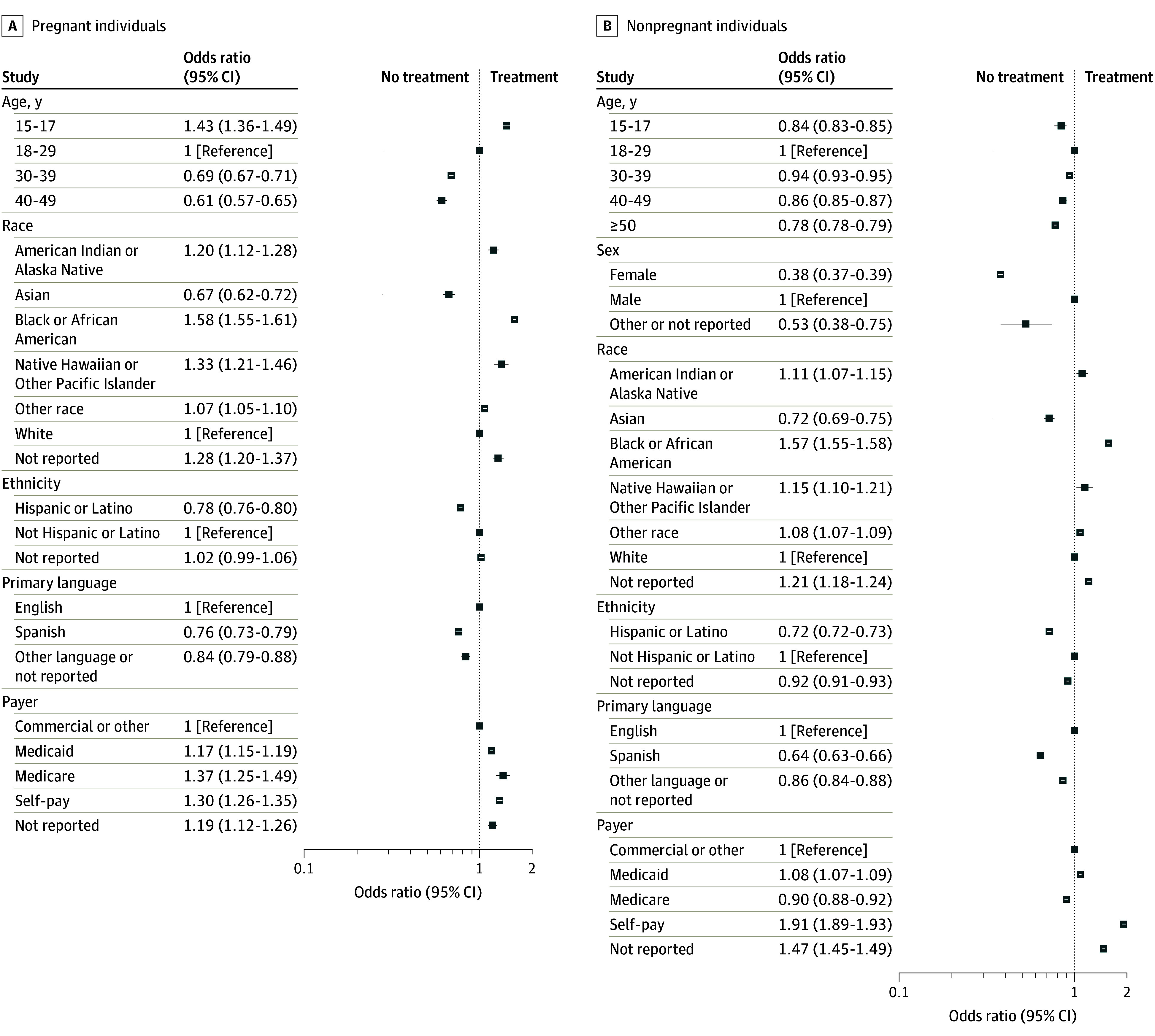
Forest Plot of Empiric Treatment Among Pregnant and Nonpregnant Patients A, Forest plot of empiric treatment among pregnant patient encounters in emergency department receiving testing for *Neisseria gonorrhea* or *Chlamydia trachomatis*. B, Forest plot of empiric treatment among nonpregnant patient encounters in the emergency department receiving testing for *Neisseria gonorrhea* or *Chlamydia trachomatis.*

Among pregnant patients, empiric treatment rates also varied by age, race, ethnicity, primary language, and insurance payer status (Figure 2A; Table). Compared with pregnant patients aged 18 to 29 years, those aged 15 to 17 years had significantly higher odds of empiric treatment (OR, 1.43; 95% CI, 1.36-1.49). Conversely, patients aged 30 years or older had lower odds of treatment compared with those aged 18 to 29 years (the reference group): those aged 30 to 39 years (OR, 0.69; 95% CI, 0.67-0.71) and those aged 40 to 49 years (OR, 0.61; 95% CI, 0.58-0.65). Pregnant patients 50 years of age or older were excluded from the multivariate analysis due to small cell sizes (≤10 treated cases). By race, pregnant patients identifying as Black or African American had higher odds of empiric treatment (13.6% [n = 26 749]; OR, 1.58; 95% CI, 1.55-1.61) compared with White pregnant patients (9.0% [n = 18 514]). Similar patterns were observed among American Indian or Alaska Native and Native Hawaiian or Other Pacific Islander patients. Pregnant patients identifying as Asian had lower odds of empiric treatment (OR, 0.67; 95% CI, 0.62-0.72). Hispanic or Latino pregnant patients had lower empiric treatment rates than non-Hispanic pregnant patients (9.1% [n = 8594] vs 11.3% [n = 37 116]; OR, 0.78; 95% CI, 0.76-0.80). Pregnant patients whose primary language was English were more likely to receive empiric treatment than those whose primary language was not English (11.2% [n = 44 117] vs 8.8% [n = 3380]; OR, 0.76; 95% CI, 0.73-0.79). Empiric treatment was more common among those with Medicaid (11.6% [n = 20 597]; OR, 1.17; 95% CI, 1.15-1.19), Medicare (13.4% [n = 453]; OR, 1.37; 95% CI, 1.25-1.49), or self-pay insurance (12.7% [n = 3859]; OR, 1.30; 95% CI, 1.26-1.35) compared with those with commercial insurance (10.1% [n = 25 863]).

**Table.** Empiric Treatment Among Emergency Department Patient Encounters Receiving Testing for *Neisseria gonorrhea* or *Chlamydia trachomatis*

Demographic characteristic	Pregnant patients		Nonpregnant patients	
	Treated (n = 49 419), No. (%)^a^	OR (95% CI)	Treated (n = 1 699 393), No. (%)^a^	OR (95% CI)
Age, y^b^				
15-17	2202 (15.9)	1.43 (1.36-1.49)	81 715 (35.3)	0.84 (0.83-0.85)
18-29	36 300 (11.8)	1 [Reference]	920 279 (39.4)	1 [Reference]
30-39	10 001 (8.4)	0.69 (0.67-0.71)	431 071 (38.2)	0.94 (0.93-0.95)
40-49	900 (7.5)	0.61 (0.58-0.65)	174 334 (36.0)	0.86 (0.85-0.87)
≥50			92 000 (33.7)	0.78 (0.78-0.79)
Sex				
Female	49 419 (12.2)	NA	929 381 (30.8)	0.38 (0.37-0.39)
Male	0	NA	769 685 (53.6)	1 [Reference]
Other or not reported	0	NA	327 (36.5)	0.53 (0.38-0.75)
Race				
American Indian or Alaskan Native	916 (10.6)	1.20 (1.12-1.28)	25 315 (33.9)	1.11 (1.07-1.15)
Asian	660 (6.2)	0.67 (0.62-0.72)	18 986 (27.0)	0.72 (0.69-0.75)
Black or African American	26 749 (13.6)	1.58 (1.55-1.61)	1 013 404 (44.1)	1.57 (1.55-1.58)
Native Hawaiian or Other Pacific Islander	481 (11.7)	1.33 (1.21-1.46)	10 722 (34.9)	1.15 (1.10-1.21)
Other race^c^	9386 (9.6)	1.07 (1.05-1.10)	239 972 (33.4)	1.08 (1.07-1.09)
White	18 514 (9.0)	1 [Reference]	586 305 (31.9)	1 [Reference]
Not reported	1583 (11.3)	1.28 (1.20-1.37)	42 742 (38.2)	1.21 (1.18-1.24)
Ethnicity				
Hispanic or Latino	8594 (9.1)	0.78 (0.76-0.80)	191 859 (32.0)	0.72 (0.72-0.73)
Not Hispanic or Latino	37 116 (11.3)	1 [Reference]	1 396 157 (39.3)	1 [Reference]
Not reported	3709 (11.5)	1.02 (0.99-1.06)	111 377 (37.4)	0.92 (0.91-0.93)
Primary language				
English	44 117 (11.2)	1 [Reference]	1 596 612 (38.8)	1 [Reference]
Spanish	3380 (8.8)	0.76 (0.73-0.79)	58 965 (29.0)	0.64 (0.63-0.66)
Other language or not reported	1922 (9.6)	0.84 (0.79-0.88)	43 816 (33.9)	0.86 (0.84-0.88)
Insurance payer status				
Medicaid	20 597 (11.6)	1.17 (1.15-1.19)	463 257 (36.9)	1.08 (1.07-1.09)
Medicare	453 (13.4)	1.37 (1.25-1.49)	34 642 (32.9)	0.90 (0.88-0.92)
Commercial or other insurance	25 863 (10.1)	1 [Reference]	863 707 (35.1)	1 [Reference]
Self-pay	3859 (12.7)	1.30 (1.26-1.35)	317 352 (49.9)	1.91 (1.89-1.93)
Not reported	1302 (11.9)	1.19 (1.12-1.26)	92 652 (45.0)	1.47 (1.45-1.49)

Abbreviations: NA, not applicable; OR, odds ratio.

^a^ Percentage of all patients in a given category who were empirically treated.

^b^ For the pregnant cohort, the patients aged 40 to 49 years and those 50 years or older were combined due to small sample sizes.

^c^ Other race is a category in Cosmos and is not further defined.

### Demographic Differences Among Nonpregnant Patients

Among nonpregnant patients, empiric treatment rates also varied significantly by age, sex, race, ethnicity, language, and insurance status (Figure 2B; Table). Treatment was most common among patients aged 18 to 29 years (39.4% [n = 920 279]). Patients younger than 15 years had lower odds of empiric treatment (OR, 0.84; 95% CI, 0.83-0.85), as did patients 50 years or older (OR, 0.78; 95% CI, 0.78-0.79). Male patients were substantially more likely to receive empiric treatment than female patients (53.6% [n = 769 685] vs 30.8% [n = 929 381]), with female sex associated with lower odds of treatment (OR, 0.38; 95% CI, 0.37-0.39). By race, empiric treatment was more common among patients identifying as Black or African American (44.1% [n = 1 013 404]; OR, 1.57; 95% CI, 1.55-1.58), American Indian or Alaska Native (33.9% [n = 25 315]; OR, 1.11; 95% CI, 1.07-1.15), and Native Hawaiian or Other Pacific Islander (34.9% [n = 10 722]; OR, 1.15; 95% CI, 1.10-1.21) compared with White patients (31.9% [n = 586 305]). Patients identifying as Asian had lower odds of empiric treatment (OR, 0.72; 95% CI, 0.69-0.75). Hispanic or Latino ethnicity was associated with lower empiric treatment rates compared with non-Hispanic ethnicity (32.0% [n = 191 859] vs 39.3% [n = 1 396 157]; OR, 0.72; 95% CI, 0.72-0.73). Patients whose primary language was English were more likely to receive empiric treatment than those whose primary language was Spanish (38.8% [n = 1 596 612] vs 29.0% [n =5 8 965]; OR, 0.64; 95% CI, 0.63-0.66). Empiric treatment was most common among patients with self-pay insurance (49.9% [n = 317 352]; OR, 1.91; 95% CI, 1.89-1.93), followed by those with Medicaid coverage (36.9% [n = 463 257]; OR, 1.08; 95% CI, 1.07-1.09), compared with patients with commercial insurance (35.1% [n = 863 707]).

## Discussion

In this large, nationally representative analysis of ED encounters involving testing for *N gonorrhea* and *C trachomatis*, we observed marked differences in empiric treatment by pregnancy status and across demographic characteristics. Pregnant patients had approximately one-fifth the odds of receiving empiric treatment compared with nonpregnant patients. Empiric treatment rates also varied by age, sex, race, ethnicity, language, and payer source.

These findings highlight the complexity of empiric treatment decision-making in ED settings, where clinicians must balance diagnostic uncertainty, concerns about follow-up, and potential risks of treatment. The substantially lower empiric treatment rate among pregnant patients may reflect heightened caution regarding antibiotic exposure during pregnancy, particularly in the absence of confirmation of a definitive diagnosis.^17^ In addition, pregnant patients often have higher rates of access to health care systems through prenatal care,^22^ which may increase clinician confidence in follow-up and reduce the perceived need for empiric treatment. However, these assumptions may not be uniformly valid, particularly for pregnant patients who rely on EDs as their primary source of care.^23^ Given the known maternal and neonatal consequences of untreated STIs,^15,16^ the observed treatment gap raises important questions about whether opportunities for prevention may be missed among some pregnant patients.

Patterns observed across payer source further illustrate how empiric treatment may be shaped by perceived barriers to follow-up. For both pregnant and nonpregnant patients, empiric treatment was most common among those with self-pay or Medicaid coverage. Centers for Disease Control and Prevention guidelines recommend empiric treatment when follow-up cannot be ensured,^14^ and higher treatment rates in these populations may reflect clinician attempts to mitigate structural barriers associated with insurance instability rather than inappropriate overtreatment.^24,25^ In this context, differential treatment may represent an effort to deliver equitable care in the face of unequal access.

At the same time, not all observed differences can be readily explained by guideline-concordant risk mitigation. The consistent finding that patients whose primary language was English were more likely to receive empiric treatment than those who had another primary language is important to note. Language proficiency does not plausibly increase biological risk for *N gonorrhea* or *C trachomatis* infection, suggesting that communication barriers may influence clinicians’ assessment of risk, symptom severity, or follow-up reliability. This pattern raises concern for inequities in care delivery that may disadvantage patients with more limited English proficiency, even within safety-net ED environments.^26,27,28,29^

Racial and ethnic differences in empiric treatment were also observed in both pregnant and nonpregnant patients, with higher treatment rates among patients identifying as Black or African American, American Indian or Alaska Native, and Native Hawaiian or Other Pacific Islander compared with White patients. These patterns parallel well-described disparities in STI prevalence at the population level^30^; however, differential treatment cannot be definitively interpreted as appropriate or inappropriate without additional context. Variable prescribing patterns may reflect clinicians’ awareness of population-level risk, differences in clinician familiarity with recommendations for empiric treatment, implicit bias, or assumptions about follow-up. Importantly, this may contribute to stigmatization if applied without individualized assessment.

Notably, this study does not establish whether empiric treatment practices are commensurate with actual STI prevalence within these populations. Without integrating treatment data with diagnostic outcomes, local prevalence estimates, or symptom profiles, it is not possible to determine whether certain groups are undertreated or overtreated relative to true risk. This limitation highlights the need for future studies that link empiric treatment decisions to confirmed infection rates and downstream outcomes.

More broadly, these findings caution against framing empiric treatment differences solely in terms of disparity. Differential treatment is not inherently inequitable. Equitable care may require different clinical approaches when patients face unequal risks and systemic barriers. However, distinguishing equitable adaptation from inequitable care requires careful attention to mechanisms, communication, and patient experience, which are factors that cannot be fully captured in aggregate EHR data alone.

### Limitations

Our study has several limitations. First, while the Cosmos dataset includes a nationally representative population, it is limited to health care systems using Epic EHR and may not reflect all US EDs. Second, the observational design limits the ability to assign causality. Third, data were not available on the *N gonorrhea* or *C trachomatis* results to inform whether empiric treatment ultimately resulted in undertreatment or overtreatment. Fourth, because gender identity data were not consistently available, we were not able to analyze differences by gender. Fifth, we cannot account for potential confounders and other explanatory factors, such as local STI prevalence, patient-reported symptoms, clinical findings, or access to follow-up that may have influenced empiric treatment decisions.^13^ Sixth, it is possible some patients received antibiotics for alternate reasons (eg, pneumonia). We were unable to account for the role of shared decision-making or patient refusal on empiric treatment.

## Conclusions

In this cross-sectional study of a nationally representative sample of ED encounters, empiric treatment for those tested for *N gonorrhea* and *C trachomatis* differed substantially by pregnancy status and across demographic characteristics. These differences likely reflect a complex interplay of guideline application, communication, and structural barriers to care rather than clinician bias alone. Aligning empiric treatment practices with clinical guidance and individual- and population-level risk may support more equitable and effective STI management in emergency care settings.

## Notes

Supplement.
Data Sharing Statement
