# Supplementary material for: Sexually Transmitted Infection Treatment Rates Among Pregnant vs Nonpregnant Patients in Emergency Departments
Source: JAMA Netw Open. 2026 Apr 15;9(4):e264911. doi: 10.1001/jamanetworkopen.2026.4911 (PMC13084434; doi:10.1001/jamanetworkopen.2026.4911)
Supplement: Supplement. — Data Sharing Statement [file jamanetwopen-e264911-s001.pdf]

Data Sharing Statement

Gottlieb. Sexually Transmitted Infection Treatment Rates Among Pregnant vs Nonpregnant Patients in Emergency Departments. *JAMA Netw Open*. Published April 15, 2026. doi:10.1001/jamanetworkopen.2026.4911

Data
Data available: Yes
Data types: Deidentified participant data
How to access data: Data will be made available from the authors upon reasonable request
When available: With publication

Supporting Documents
Document types: None

Additional Information
Who can access the data: Researchers whose proposed use of the data has been approved
Types of analyses: After approval of a proposal
Mechanisms of data availability: With a signed data access agreement
